# Documenting genomics: Applying archival theory to preserving the records of the Human Genome Project

**DOI:** 10.1016/j.shpsc.2015.08.005

**Published:** 2016-02

**Authors:** Jennifer Shaw

**Affiliations:** Wellcome Library, 215 Euston Road, London, NW1 2BE, UK

**Keywords:** Human Genome Project, Archival theory, Scientific collecting

## Abstract

The Human Genome Archive Project (HGAP) aimed to preserve the documentary heritage of the UK's contribution to the Human Genome Project (HGP) by using archival theory to develop a suitable methodology for capturing the results of modern, collaborative science. After assessing past projects and different archival theories, the HGAP used an approach based on the theory of documentation strategy to try to capture the records of a scientific project that had an influence beyond the purely scientific sphere. The HGAP was an archival survey that ran for two years. It led to ninety scientists being contacted and has, so far, led to six collections being deposited in the Wellcome Library, with additional collections being deposited in other UK repositories. In applying documentation strategy the HGAP was attempting to move away from traditional archival approaches to science, which have generally focused on retired Nobel Prize winners. It has been partially successful in this aim, having managed to secure collections from people who are not ‘big names’, but who made an important contribution to the HGP. However, the attempt to redress the gender imbalance in scientific collections and to improve record-keeping in scientific organisations has continued to be difficult to achieve.

## Highlights

• The Human Genome Archive Project aimed to preserve scientific records.
• Previous approaches to scientific collecting were not suitable for big science.
• Archival theories were assessed before documentation strategy was chosen.
• The project has led to new collections being deposited in UK archives.
• Problems still remain with scientific record-keeping.

When citing this paper, please use the full journal title *Studies in History and Philosophy of Biological and Biomedical Sciences*

## 1 Introduction

The failure to preserve the records of scientific endeavour is intermittently bemoaned by scientists, historians and archivists. In some cases, this is followed by activity to try to remedy the situation. While American geneticist L. C. Dunn was writing his *Short History of Genetics* he became concerned about the loss of material due to the: “Failure of working scientists to preserve their correspondence and other unpublished papers and to bequeath them to an appropriate repository” (Dunn, 1965). Dunn led by example by depositing his papers at the American Philosophical Society and actively encouraged other scientists to do the same.^1^

Similar concerns to those expressed by Dunn can also be seen from the archival perspective, with the lack of training and interest shown by archivists in scientific material both raised as issues. In 1969 during his presidential address at the Society of Archivist's annual general meeting Roger H. Ellis stated that the records of science and technology: “Do not at present figure largely in our holdings, and up to now we have not been taught anything specific about them in our Diploma Courses” (Ellis, 1970, p. 87). Despite Ellis's hope that there was the prospect of change for the latter, the records of science and technology still do not feature as a significant aspect of any of the post-graduate training courses for archivists in the UK and Ireland. There are currently seven universities offering record-keeping courses accredited by the Archives and Records Association (UK and Ireland).^2^ The majority of modules on these courses focus on the theoretical basis of record-keeping or the key skills required in this area, such as managing digital records or palaeography. The need to equip archivists with the necessary skills for an evolving workplace, in particular the need to manage records in both analogue and digital formats, has seen a gradual reduction in the number of subject specific modules and a greater emphasis on the overarching principles that apply across the management of archives. Although now in the minority, a few subject specific modules remain on corporate records, ecclesiastical archives, education archives, house history and military archives. The records of science and technology still do not feature on the post-graduate courses and, unless there is a reversal of the current trend, a dedicated module is unlikely in the foreseeable future.

Against this backdrop of concern that not enough was being done by all sides to preserve the records of science and technology in general, in the late 2000s key organisations involved in the Human Genome Project (HGP) started to worry about these records specifically. In part this was prompted by the retirement of key personnel, but also the format of the records. The era in which the HGP happened meant that a large proportion of the primary material was created in born-digital format making it more vulnerable to loss than its analogue equivalents. In response to this concern, a meeting was held at Cold Spring Harbor Laboratory, New York in 2009 to discuss the importance of the HGP, raise awareness amongst scientists and ascertain what action needed to be taken to preserve its documentary heritage.^3^ The initial idea was for a single, international project to locate and preserve records relating to the HGP, but it quickly became clear that the logistics and funding for this type of project were impractical. Instead, it was decided that national projects would be established to undertake the majority of the work in each country whilst maintaining close collaboration and communication with each other. The aspiration remains to unite the work of these national projects in the future. The UK project, known as the Human Genome Archive Project (HGAP), was launched by the Wellcome Library, funded by the Wellcome Trust, in January 2012 to preserve the documentary heritage of the HGP in the UK.^4^ This paper will tell the story, from an archivist's perspective, of how archival theory was applied to modern collaborative science to develop and implement a suitable survey methodology by the UK project between January 2012 and December 2013.

## 2 Approaches to scientific collecting

The first question that the UK project faced was; where do you start when trying to survey contemporary, collaborative science? The most obvious starting point appeared to be a review of how scientific collecting had been approached in the past. This work mainly focused on the UK because the archival landscape can vary greatly from country to country, often influenced by legislation and funding mechanisms. However, some approaches from overseas were also investigated, but only in Anglophone literature.

There has been regular activity in the UK to preserve the records of individual scientists since the early 1960s. The majority of this work can trace its origins back to when Lord Evershed, chairman of the Royal Commission on Historical Manuscripts (HMC), asked Commissioner Roger Quick to form a plan for addressing what he saw as a neglected area in the UK's archive provision. This eventually led to the establishment of a pilot project organised by the Royal Society and the HMC in 1967, which surveyed three distinguished and recently deceased Fellows of the Royal Society, namely pharmacologist and physiologist Sir John Gaddum, physicist Sir Francis Simon and geologist Professor Lawrence Wager.^5^ Their records were collected, sorted, listed and deposited with the Royal Society's library.^6^ The project successfully demonstrated tangible results with limited resources. It also set a number of precedents that have been applied to scientific collecting in the UK ever since.

Prior to the pilot it had been imagined that a physical archive would be established for scientific collections, but as a result of the pilot this changed. Instead, the idea for a processing centre was proposed where collections would be collected and catalogued before a suitable permanent archival home could be found for them. The idea of operating a processing centre had many aspects in its favour, but a key benefit was that it would be much more economical to operate as it would lack the burden of long-term storage costs. As a result, in 1973 the Contemporary Scientific Archives Centre was established in Oxford. This was initially a three year trial with the Royal Society acting as the main financial supporter (Gowing, 1979). Over the years the processing centre evolved with changes in funding, staffing and location, but the method of working remained stable. In 1987 the Centre moved to Bath and became known as the National Cataloguing Unit for the Archives of Contemporary Scientists (NCUACS). The Unit continued to collect scientific papers, catalogue them and then find a suitable archival home for them until 2009 when the Royal Society withdrew its funding and the Unit was forced to close.^7^ Following the closure of the NCUACS, the Centre for Scientific Archives was established as a processing centre for scientific collections based at the Science Museum's site in Wroughton, near Swindon.^8^ Its work is broadly similar, but it does not have any core funding; instead it receives educational grants for the cataloguing of specific collections.^9^

There has certainly been a high level of continuity in the approach used to preserve scientific archives in the UK with the methodology developed as part of the pilot in the 1960s still being applied nearly fifty years later. This approach has undoubtedly yielded tangible and positive results. It also achieves these results in a highly economical way with relatively low resource requirements. Although it takes time to catalogue archive collections, by far the biggest costs are the long-term care and storage of these collections and providing access for researchers, which this approach negates the need for. By not collecting material itself the pilot scheme set a precedent for material going to the most appropriate repository, fostering a culture of co-operation rather than competition among archives. Although as bemoaned by Ellis, scientific collections were still absent from theoretical archival training, the processing centres were giving archivists practical experience of preserving the papers of outstanding scientific individuals.

The number of scientific collections in UK archives undoubtedly increased as a result. The NCUACS catalogue proudly points to over 250 collections that not only exist, but are catalogued and can be made available for research as a result of their work (Powell & Sheppard, 2006, p. 460). Many archivists pay tribute to the work of NCUACS and its successor as fulfilling a vital role in processing collections that they do not have the resources to undertake themselves. NCUACS also performed a wider role, that of raising the profile of scientific collections.

However, there are significant drawbacks to this method of collecting. Firstly, let us examine the practice of reactive collecting after the death of the creator. This is a very common method of acquisition for archives and is certainly not confined to scientific collections. The perpetuation of this practice is in part due to the limited resources that most archive services operate with, but many have argued strongly against it in the archival literature (Cox, 1994, Ham, 1975, Samuels, 1992, Zinn, 1977). These calls for archivists to be more proactive in their collecting date from a period when archivists dealt with mainly hard-copy material. The more recent need for archivists to manage records in born-digital format, records whose original format is digital as opposed to hard-copy records that have been digitised, has served to exacerbate the problem.^10^ As the volume of digital material being produced in our working and personal lives has increased so has the volume of digital material that makes its way into archive collections which is eroding the viability of the passive approach to collecting. Natalie Ceeney, writing while chief executive of the UK's National Archives, expressed it thus: “The notion that we can wait 30 years and receive digital files without active intervention is laughable. We cannot assume that records will be kept unless destroyed, the opposite is true” (Ceeney, 2008, p. 65). Indeed all of the guidance surrounding digital preservation is that these records are far more vulnerable to loss than their hard-copy equivalents and action needs to be taken sooner rather than later if they are to survive for future generations.^11^ The costs involved in digital preservation can also become prohibitive as time is allowed to elapse. Although many of the dire warnings regarding software obsolescence have not yet come to pass, many archives, like the Wellcome Library, have already experienced problems with hardware obsolescence. Archival best practice states that for successful digital preservation archivists really need to engage with the record creators directly and with the material as soon as possible^12^ and our experience would support that advice.

A second drawback to the traditional method of collecting was its focus on collecting the papers of outstanding individuals, such as Nobel prize winners or Fellows of the Royal Society, which can give the impression that science is an individual pursuit; the work of a lone genius. Again, this is not necessarily a collecting problem solely related to science. Zinn argued strongly that too much attention was paid to the important and powerful in all areas of society while the ordinary were largely ignored (Zinn, 1977). In science the lone genius may be true in some limited cases, but much of modern science, particularly Big Science, is inherently collaborative (Galison and Hevly, 1992, Lenoir and Hays, 2000, Parker et al., 2012). The HGP involved high levels of co-operation and collaboration across institutional and international boundaries, despite the fact that most of the media attention was focused on the competition element of public versus private.^13^ Taking the sequencing of one of the smallest chromosomes as an example, chromosome 22 involved collaboration between teams in the UK, Japan, the US, Canada and Sweden.^14^ The publication of the gold standard human genome in 2004 lists 745 authors from the Sanger Institute alone, just one centre in the public consortium (International Human Genome Sequencing Consortium, 2004). Clearly collaborative science projects, like the HGP, encompass large numbers of people and institutions and the traditional approach of focusing on outstanding individuals does not adequately capture their work.

The third drawback is how the focus of scientific collecting has tended to be on individuals rather than organisations.^15^ In fact very little has been done to address the lack of systematic record-keeping in scientific institutions or to preserve the records of these organisations. The Royal Society/HMC pilot scheme had defined and limited terms of reference. When establishing the pilot scheme, a conscious decision was made to concentrate on personal papers as it was believed that this would be more straightforward to achieve and provide more tangible benefits. Institutional records were placed to one side for what was intended to be the time-being, but which has unfortunately been a protracted time-being (Ellis, 1970, p.93). In the course of surveying for the HGAP I have found that while some scientific institutes are bursting at the seams with historical material, others appear to have destroyed most things. In the field of physics there is generally better provision for archives than the field of biology. Several of the large physics facilities, notably SLAC National Accelerator Laboratory and CERN, have in-house staff and policies on the retention of their records.^16^ The lack of a record-keeping culture within many scientific establishments means that the scientists who pass through them are not given training in this area nor can they see the benefit of it. It can be hoped that the recent decision by EMBL to recruit an archivist might improve the amount of training in record-keeping that scientists receive. EMBL's mission “to promote molecular biology across Europe, and to create a centre of excellence for Europe's leading young molecular biologists” is an opportunity to raise awareness of scientific archives to each generation that passes through.^17^

Although resource efficient, the establishment of an organisation that processed scientific collections, at minimal inconvenience to the archive repositories that were eventually to house the collections is an additional drawback, which has meant that scientific collecting has not become embedded within individual repositories. For many archivists science has generally been seen as a problem that someone else will deal with. It has also kept knowledge of working with scientific collections within a very small part of the archives sector. The closure of NCUACS in 2009 has caused some repositories to reconsider their approach to scientific collections. Rather than leaving the responsibility of scientific collecting to another organisation, several archives have increased their activity in this area. One particularly proactive example is the Bodleian Library's Saving Oxford Medicine project which was launched in 2011 to process collections already held so that they could be made available for research and also to survey material still in the hands of its creators.^18^

Finally, the priority of the traditional approach to scientific archives has always been cataloguing rather than collecting. The lack of an overarching strategy for collecting meant that this was not really a model that could be successfully applied to the HGP. Although what had previously been done for scientific collecting preserved important collections and established a link between science and archives, it was unable to provide a suitable methodology for the HGAP, so it was clear that an alternative approach was necessary. This led me to consider archival theory and what solutions it could bring to the problem.

## 3 Archival theory

Just as historiography has evolved over the years (de Chadarevian, 2016) so has archival theory with emphasis shifting in response to the challenges faced by each generation of archivists and the perceived failures of their predecessors. As Ceeney (2008, p.69) neatly summarises it: “The paradox being that whilst 60 years is nothing for an archival document, it's a very long time for archival thinking.”

In 1922 the first edition of Hilary Jenkinson's *A Manual of Archive Administration* was published based on his experience in the Public Record Office, now known as the UK's National Archives. This text remains a feature of archival post-graduate teaching in the UK nearly a century after it was written. Jenkinson believed that the archivist should be a passive curator of the past and should not make selecting decisions; instead these should be left to the creator:

“In fine, for the Archivist to destroy a document because he thinks it useless is to import into the collection under his charge what we have been throughout most anxious to keep out of it, an element of personal judgement; for the Historian to destroy because he thinks a document useless may be sager at the moment (since he presumably knows more history than the Archivist), but is even more destructive of the Archives' reputation for impartiality in the future: but for an Administrative body to destroy what it no longer needs is a matter entirely within its competence and an action which future ages (even though they may find reason to deplore it) cannot possibly criticize as illegitimate” (Jenkinson, 1922, p. 128).

Jenkinson's view of collecting and appraisal was strongly influenced by the scarcity of individual manuscripts from the medieval and early modern periods. While his goal of objectivity has endured, much of his theory has been superseded in the face of the bulk of modern records and whether his approach actually achieves objectivity. As Jarman (2012, p. 47) states: “The emergent view suggests that the Jenkinsonian ideal of a passive record-keeper can result in collections that are just as biased as those that have been selected on the basis of assigned value.” If the Jenkinsonian theory for archives was already starting to crack under the strain of the volume of modern hard-copy records, the advent of digital records has demolished it as a practical approach.

For the HGAP, an archive project that was consciously trying to collect proactively, this was clearly not an appropriate approach to take. However, it is still possible to find some value in it. Jenkinson's emphasis on the importance of the record creators is a useful concept for this project. For any archivist needing to work with a highly specialised collection outside of their area of expertise the ability to harness the expertise of the creator is invaluable, even if caution is needed. It can be difficult for record creators to recognise the wider significance of their work or their collections so in this area the judgement of archivists and historians, often working together, is essential. However, for identifying and qualifying material the creators are vital. Although not something advocated by Jenkinson, for much of our modern collecting the archivist seeks to develop a close relationship with the creator, be that individual or organisation, to identify relevant material and to be available for clarification during processing. For instance, the recently released catalogue for Alan Coulson's archive collection (Wellcome Library reference PP/COU) was catalogued by an archivist with input from the creator. The cataloguing archivist was able to discuss with Coulson how to arrange the material so that it accurately reflected how the material had originally been created and used. As a result the material relating to genome mapping was not separated from material relating to genome sequencing as this was not a fair representation of how the work was carried out.^19^

Having judged Jenkinson's approach inappropriate for the HGAP, I looked at what alternatives could be offered by archival theory. Another doyen of archival theory is Theodore Schellenberg, who published his book *Modern Archives* in 1956. Schellenberg proposed a much more active approach to archive collecting than Jenkinson. He placed much more emphasis on the users of records rather than their creators: “The archivist is usually an historian by training, and, as a matter of course, will preserve records containing evidence of the development of the government and the nation that is valuable for historical research…He is familiar with research needs and interests” (Schellenberg, 2003, p. 30). Although, this level of prominence attached to the end-users will seem attractive to many researchers it must be applied with caution. It is dangerous to try to predict the uses of records and collect based on these. Just as approaches to archives have changed over time, so have historical approaches and collecting too tightly based on the interests of today undermines the long-term prospects of a collection.^20^ However, an awareness of research approaches is useful for the archivist in thinking beyond the narrow genre of biography (Aicardi, 2016). Current research approaches have certainly been useful to justify collecting decisions such as being able to demonstrate the importance of records about the construction of the Sanger Centre based on interest in the spaces where science is done and retaining file series from meetings that scientists attended in their collections, such as the extensive files from scientific meetings included in the Michael Ashburner collection (Wellcome Library reference PP/MIA), to show how scientific networks developed.^21^

Whilst Schellenberg's record users might not be at the forefront of our collecting decisions anymore, the concepts he proposed about the differing values of records have been influential in this project. Schellenberg put forward the concepts of informational value and evidential value which have been a useful touchstone when trying to capture the records of ground-breaking science (Schellenberg, 2003, pp. 140–160). Informational value relates to the content of the material whereas evidential value relates to the process by which the material was created, thus providing contextual information about the creating organisation or individual. The survey project has also led to a re-evaluation of the type of material that is contained within a scientific collection and Schellenberg's values have been a useful concept in this area. When considering the records of science, particularly laboratory notes or results of experiments it is not the informational value that is the most important. Rather it is the evidential value that is paramount. As techniques change, and in developing fields such as genomic sequencing this change has been rapid, it can be just as important to capture evidence of a technique before it becomes obsolete.

Although the theories of both Jenkinson and Schellenberg had some useful elements for the HGAP, they could not provide the conceptual approach that was needed. The next theory I considered was macroappraisal, a top-down strategic approach associated with Terry Cook. Macroappraisal is based on an assessment of how the record was created and places a greater emphasis on why, where and how records were created rather than the actual information that is contained within them. In practical terms, it changes how archivists assess which records will be retained (Cook, 2005). Whereas with previous techniques an archivist would go through material file by file or volume by volume, with macroappraisal the archivist considers what material should be kept at a more theoretical level and then applies this to the material in hand. To a certain extent macroappraisal is a response to the expanding volume of documentation during the latter part of the twentieth century, something that Jenkinson did not have to contend with. It is an attempt to help the archivist to make decisions at a higher level so that systems can cope with the volume of material being processed.

The macroappraisal approach has been used by a number of national archives to produce strategic vision and coherence with notable examples being the national archives of Canada, Australia and the UK. The UK's National Archives states its approach thus:

“Macro-appraisal encourages government-wide or organization-wide analysis of functions as a guide to identifying records of value for business or archival purposes. It may be appropriate for digital records because, by identifying records produced by the most significant functions, it provides the means to make appraisal decisions without the need for file-by-file scrutiny or the ‘historical perspective’ provided by the passage of time.”^22^

As the examples of national archives show, macroappraisal has generally been applied within an institutional setting where there are a number of common factors: the records have already been identified; there is a clear route for their transfer to an archive; and there is a legal mandate for transfer, as is commonly the case for the records of central government and their appointed place of deposit. This is solving a very different problem to that facing the HGAP and has taken place in a very different organisational setting. Macroappraisal is often applied where the volume of records is large, but the essential problem of the HGAP was not processing a large volume of records that were already known to exist; rather it was primarily concerned with locating those records in the first place. I also questioned the extent to which it could be applied beyond administrative records, which are much more regular and easier to predict than the records of individual scientists or research teams. It should be possible to apply macroappraisal to records generated by a single scientific institution, whose functions, such as scientific administration or public engagement, and records series, such as minutes from scientific advisory group meetings, should be straightforward to identify. However, I doubted that it could work for the personal records of individual scientists that are generally much less structured and predictable. Therefore, although macroappraisal could assist with some of the appraisal decisions needed during the HGAP, such as when deciding which record series should be kept, I did not feel that it was a particularly appropriate way of addressing the problem.

Finally, I looked at documentation strategy, a theory that became increasingly written about during 1980s and is closely associated with Helen W. Samuels (1986). It is described as “A methodology that guides selection and assures retention of adequate information about a specific geographic area, a topic, a process or an event that has been dispersed through society” (Pearce-Moses, 2005, p. 131). A theory based on the complex interaction of many different record creators and relating to something that was dispersed rather than centralized in a single institution instantly appeared to be the type of guiding principle that the HGAP needed. In particular, there were a number of aspects to documentation strategy that made it feel particularly appropriate for the HGAP. Firstly, documentation strategy aims to look beyond institutional frameworks, which would help assist the HGAP in considering many types of organisations as well as individuals. Secondly, it was based on the idea that the standard institutional approach was too narrow and piecemeal which would be beneficial in helping the HGAP to look beyond a single person or organisation to try to provide a coherent picture. Finally, it was based upon the concept of looking broadly at an event which impacted more widely through society, which would allow the HGAP to not treat the science as happening in a vacuum; instead it could embrace its wider interaction with ethics, politics, technology and society.

## 4 Documentation strategy and my approach to the HGAP

Documentation strategy was eventually chosen as the approach for the HGAP because it offered a broad overarching concept that would help to guide the project, but which could be applied flexibly to take reality into account. However, the implementation of documentation strategy has had mixed results and it does have its critics within the archive community. Doris J. Malkmus's evaluation of five archive projects that have attempted to use documentation strategy highlights some of the pitfalls, but also draws out factors that can aid success (Malkmus, 2008). These problems included both the practical, such difficulties in collaborating with other repositories that have very different collecting policies and the cost of running projects like this, as well as the conceptual, such as how it is unrealistic to try to document all human activity even in a defined area. Amongst the factors for success Malkmus includes some that are clearly beyond the control of the archivist, such as there already being a well established community in the subject area or the presence of familiar sources whose existence it is possible to predict. Nevertheless, these are still important aspects to consider before embarking on a project based on documentation strategy. However, other success criteria are within the control of the archivist such as crafting a well-defined topic and establishing a good advisory group. These elements have all been important factors in the application of documentation strategy for the HGP. An additional benefit has been having the project based at a credible host institution, the Wellcome Library, to generate the trust required to pursue the project. I would add to these success criteria the importance of a healthy dose of pragmatism when using documentation strategy. Although aiming to document all human activity in a given area is unrealistic, when used as a philosophical approach or guiding principle documentation strategy is a useful way of thinking broadly about an area of activity and then focusing in to capture what is important and feasible.

One institution that has successfully applied documentation strategy over several projects is the American Institute of Physics (AIP) and it has done so in an area that is particularly relevant to the HGAP. In 1989 the AIP began its work to preserve records relating to multi-institutional collaborations involved in big physics. They applied documentation strategy for their first project to capture the dispersed records of multi-institutional collaboration in the subject of high-energy physics (Warnow-Blewett, Maloney, & Nilan, 1992). As part of this survey for high-energy physics they identified particular areas of interest that they wanted to capture, such as significant discoveries and the difference in funding at certain sites (Warnow-Blewett et al., 1992; Section VI C). The AIP project benefitted from the fact that the major physics laboratories all had record-keeping staff in-house, something that is still largely absent in biology laboratories.^23^ The AIP tends to focus its collecting work on orphan collections, collections that do not have a natural archival home, and then works closely with other archive repositories to direct relevant material into their collections. The AIP found this approach useful and successful so repeated it for later projects in space science and geophysics (Warnow-Blewett, Capitos, Genuth, & Weart, 1995) and ground-based astronomy, materials science, heavy-ion and nuclear physics, medical physics, and computer-mediated collaborations (Warnow-Blewett, Genuth, & Weart, 1999).

Although documentation strategy provided a good philosophical approach for the HGAP, the project still needed a roadmap for implementation. It found this in the Minnesota Method: “An (sic) strategy for appraising materials that combines aspects of collection analysis, documentation strategy, appraisal, and functional analysis.” (Pearce-Moses, 2005, p.253). The methodology was created by the Minnesota Historical Society for business records in a specific geographic area so needed to be slightly adapted before being applied to the records of collaborative science (Greene & Daniels-Howell, 1997).

The Minnesota Method is a multi-staged process that has been adapted and applied for the HGAP in four stages. The first stage for the HGAP was to define the collecting area. The project approved by the Executive Board of the Wellcome Trust provided a geographical and chronological framework that the HGAP would cover the UK and the period 1977–2004. A decision was made early on that where careers extended beyond the scope of the collecting area this material would still be included. For instance if a scientist who was closely involved in the HGP had a career that included time working outside of the UK this material would still be surveyed, equally if a scientific career started in 1967 this early part of their career would still be surveyed so that the coherence of collections could be maintained. The finer boundaries of the project were gradually defined in tandem with setting priorities. Sometimes the existence of other archive projects influenced these priorities. For instance, a lot of the important work done by medical and human geneticists was not prioritised by the HGAP because this area of work had already been surveyed by NCUACS in November 2008 followed by a human genetics archives project based at Special Collections and Archives at Cardiff University after the closure of the NCUACS.^24^

The second stage for the HGAP was to analyse existing collections. This was an important step in the early stages of the project, which helped to establish the current archival situation. A range of catalogues, aggregated catalogues and national indexes were consulted^25^ to establish if any of the key individuals had already deposited their material in archive repositories. It also provided a good opportunity to see if any of the key institutions involved already had good record-keeping procedures in place. I also started to build relationships with other archive repositories that might have relevant collections or might be interested in acquiring collections in this area by investigating their collecting policies. The intention of the HGAP was to continue the practice of co-operation within the UK's archival landscape by attempting to secure the deposit of collections in the most appropriate repository. It was during this stage of the project that useful links were established with other archives, such as those at the Bodleian Library and the University of Edinburgh.

The third stage was to “determine the documentary universe including relevant government records, printed and other sources” (Pearce-Moses, 2005, p.253). For the HGP I used this stage of the project to help place the scientific records within their broader context, to capture how the HGP was influenced by external factors and how it influenced them in turn. Some of these areas and types of records already have good mechanisms for preservation in place. In these cases I wanted to record some basic information, but keep the focus on those records that have a less well-established means of preservation. The HGP was an unusual scientific project in terms of the level of political involvement; not many scientific achievements are announced by the President of the United States with a video link to the UK's Prime Minister.^26^ In the UK there is already a well-established archival route for most political records. The records of government departments are preserved by The National Archives; records of parliament are preserved by the Parliamentary Archives; and politicians, particularly former Prime Ministers, tend to make arrangements to deposit their collections soon after retirement from politics. The most complicated work that was required in this area was tracking the changing names and remits of departments.^27^

The ethical questions raised by the HGP were another area of interest. However, much of the debates and concerns in this area reached beyond the human genome to cover human genetics and genomics more broadly and often even broader still to cover bioethics. This broader remit risked taking the project off course so a conscious decision was made to ensure that the Wellcome Library had good collections of publications and grey literature in this broader area by working with librarians. This approach of broadening the type of sources that archivists consider for preservation already has supporters within the archival community (Cox, 1994, p. 14). It has also demonstrated possibilities for preserving the activity of an organisation when we might not wish to collect their archives or they might not be in a position to deposit them. In particular it might be appropriate for preserving material on the activity of biotechnology companies and their products where concerns over commercial confidentiality might limit their willingness to deposit records or allow public access. It is similar to an approach advocated by Samuels when she looked at the records of large numbers of railway companies in the US. She suggested conducting an evaluation of published sources and recommended preserving a minimum archival record (Samuels, 1986, p.121). Such an approach might be beneficial for archivists, but will undoubtedly have an impact on researchers. This approach was not applied for the HGAP, but is being considered for future collecting projects involving genomics.

One area where it was difficult to survey was that of pressure or interest groups. Due to their often transitory nature and small size they can be difficult to identify, particularly a number of years later when they are no longer active. In this area it was felt that the Wellcome name could be a hindrance, with groups sceptical about our intentions. We considered working with other archive repositories that already collect the records of this type of person or organisation, such as the Bishopsgate Institute, but so few groups were identified as to not make the process worthwhile for the HGAP.^28^

The fourth, and for the HGAP final, stage was to prioritize areas of collecting. The original Minnesota Method has additional stages, but these were felt to not be particularly relevant to the HGAP so were excluded as they were more suited to the business focus that the methodology was originally designed for.^29^ In terms of prioritization, although the project aimed to place the HGP within its wider context, the scientific material remained the main focus. One of the original drivers for the project was the concern that these records were at risk as most were still with their creators and there were insufficient established routes into archive repositories. This final stage of the project started with research into the individuals and organisations whose work related to the HGP in as broad a sense as possible. An attempt was then made to identify overarching themes that were particularly significant for the HGP, such as the growth of automation in the whole process of sequencing. Despite the fairly Whiggish chronological scope of the project, from the successful development of Sanger sequencing to the successful publication of the human genome, an attempt was made to move away from a purely Whiggish interpretation of the HGP.^30^ This included trying to find records of technology that did not work, or that was not particularly successful. Although much of the machinery that was unsuccessful leaves few artefactural traces because they were often dismantled for reuse some records were discovered during surveying, such as photographs of the machines in use or presentations explaining how they would, or should, work.^31^

From the broad scope and large numbers involved in this initial research the next task was to reduce this down to a level that would be realistic for the HGAP; that is one archivist working on a twenty-four month project. In terms of projects that have applied documentation strategy this one is relatively modest. Large groups of individuals and organisations were excluded for a variety of reasons. Some were excluded because their link to the HGP was small and formed only a limited part of their work. Others were excluded because of other archive projects that had already been completed or were in progress.^32^ As already mentioned the NCUACS had already conducted a survey into medical genetics so this area was excluded. In consultation with the project advisory group three important scientific survey areas that were significant for the HGP in the UK were identified along with those individuals and organisations that made a significant contribution to each area. Within each area four levels of priority were identified, designated A to D. The aim of this was to ensure that activity across the whole survey period was as even as possible. There was also a very practical reason. By breaking down the project into manageable pieces it was hoped that momentum could be maintained and progress measured.

The first of the identified scientific survey areas involved relevant work that took place at the Medical Research Council's (MRC) Laboratory of Molecular Biology (commonly referred to as the LMB) in Cambridge. The main priorities in this area for the HGAP were: the work of Fred Sanger and his teams in developing sequencing techniques; work on the model organism *Caenorhabditis. elegans* and how this developed into the worm genome project; and the early software programs that were developed for storing and analysing sequence data. The LMB has its own archive, but its collecting remit is only for organisational records rather than collections from individual scientists who have worked there. However, the archivist has helped many scientists over the years to find a suitable repository.^33^ This meant that it was primarily individual collections that were of concern for this area as systems were already in place to capture the organisational records.

Each of the three scientific survey areas presented different archival challenges. This area had the advantage of an institutional archive, but the passage of time since was a significant problem. In the intervening years many archival risk points will have been encountered, such as office moves or retirement, when records are likely to be destroyed. However, it was relatively straightforward to locate most of the people of interest as many had not moved far from Cambridge after retirement even if many of them had already disposed of what material they had.

The second scientific survey area looked at the setting up of the Human Genome Mapping Project in the UK which involved an MRC funded resource centre to supply necessities to laboratories embarking on mapping and sequencing work. It also involved a series of programme grants which were available for pilots and proof of concepts. Most of the successful applicants were based in UK universities or MRC units. This stage also included the UK's contribution to the establishment of the Human Genome Organisation (HUGO) in 1988.

This area caused the most concern for the HGAP. Although the MRC has record-keeping procedures in place these don't always extend out from head office to its research units as effectively as would be liked. Also while MRC head office regularly transfers records selected for permanent preservation to The National Archives, these tend to be policy and high-level records rather than the records of the actual scientific work.^34^ In terms of the universities, it is a very varied picture across the UK. All of the universities of interest for this project have established archives, but several of these repositories do not include science or technology as part of their acquisition policy. As for HUGO, its lack of a stable home has seen regular office moves over the years which have proved risky for the archives. What records remain are only a small proportion of what one would hope to find.^35^ Although the survival of organisational records for this area is disappointing, there is good material to be found in the collections of particularly active individuals, such as Sir Walter Bodmer.^36^

The third, and final, scientific survey area looked at the establishment of the Sanger Centre in 1992 and activity on the Genome Campus in Hinxton, just outside Cambridge. This represented a serious investment by the MRC and the Wellcome Trust to ensure that the UK continued to play a significant role in the global sequencing effort. One of the major challenges for this survey area was the large numbers of people involved and the fact that many of them still have active careers, leaving them little time for thinking about their archives.

The HGAP consciously aimed to not focus solely on senior scientists, but it was apparent that it was necessary to strike a careful balance and not go so far the other way as to exclude them from the survey. It was decided, in consultation with the project advisory group, to start with the Sanger Centre's, now the Wellcome Trust Sanger Institute, Board of Management and chromosome team leaders before moving down the hierarchy to try to survey as many scientific activities as possible. The initial plan for this stage was equally interested in staff based at the European Bioinformatics Institute (EBI), an outstation of the European Molecular Biology Laboratory (EMBL) which is co-located on the Genome Campus with the Sanger Institute. However, EMBL's plans to establish their own archive meant that the number of EBI personnel included in this survey stage was reduced and no collections from scientists based entirely at the EBI have been deposited yet. This stage also sought to look beyond the purely lab-based work of the scientists to capture a more rounded picture of those involved and show some of the culture and community of the site, such as the annual pantomime that was a feature of the HGP in the UK (Sulston & Ferry, 2002, pp. 237–238).

During the course of the HGAP other archive repositories have added relevant new collections to their holdings, such as the Bronwen Loder collection acquired by the Bodleian Library in 2013.^37^ To date, the Wellcome Library has taken in six collections from individual scientists as a direct result of the HGAP, but more are expected in the future as negotiations are still in progress with several other scientists. Of these deposited collections, those of Carol Churcher, Richard Durbin, Matthew Jones and Sir John Sulston have already been catalogued and are available to researchers. The remaining collections, those of Michael Ashburner and Ian Dunham, have been scheduled for cataloguing and will become available during 2015.^38^

## 5 Conclusion

One of the criticisms that documentation strategy has faced is the amount of resource required to implement it, resource that archives often do not have. The HGAP was made possible thanks to dedicated project funding from the Wellcome Trust. For UK archives this was an unusual project, rather than allocating resources to the processing of existing collections it has instead been allocated to try to locate new collections, something actually advocated by Ham forty years ago (Ham, 1975, p.13). Still, it must be acknowledged that there is a definite risk in allocating resources to projects like this. It can be difficult to quantify and demonstrate the success of survey projects with only small tangible benefits at the end. However well the project is planned, not all of the people contacted will reply. Even fewer will still have any material and a very small proportion will have material that is suitable for deposit in an archive, assuming that they are willing to deposit it. During the course of the HGAP, ninety people were contacted and of these sixty-four replied. The original aim had been to achieve a response rate of fifty per cent so we were pleased with our result of seventy-one per cent. Of those who replied, seventeen had some material which was surveyed and six then deposited this material in the Wellcome Library, while some collections were deposited or promised to other archives.

It can also be difficult to run initiatives like this as projects because a significant period of time can elapse for each stage of the process. Archivists are well prepared for the long-haul as it is not uncommon for relationships with potential donors to be measured in years, in some cases decades, rather than months. This can make this type of project seem like very poor value for money compared with a cataloguing project, which can deliver tangible results in a much more predictable timeframe. It also creates a dilemma because although you might need specific project funding to be able to undertake an initiative based on documentation strategy, there is an awareness that it does not fit neatly into the time-limited bounds of a project. We, therefore, aimed to ensure that the HGAP had an impact on our work beyond the narrow confines of the HGP. Ultimately, we have used it to reinvigorate our approach to collecting across our whole remit of health and to reconsider how we articulate our collecting decisions.

In our attempt to do something different we have been partially successful. The HGAP has seen us start conversations with scientists much earlier in their working lives than was previously the case and this has yielded benefits. All of the conversations with scientists who retired over five years ago yielded no new collections for archive repositories with the main reason being that they had already disposed of their material. Whereas, all six collections acquired by the Wellcome Library came from scientists whose careers are still active, or at least semi-active. We were also attempting to redress the balance of collecting in science so that we did not just take collections from the ‘big names’. We had some success in this area, but it has proved to be challenging. Senior scientists are more likely to work in conditions that are conducive to the survival of their records, namely they will have an assistant to help with paperwork and the space to be able to store this material. This may continue even after the scientist has left the organisation. As a result these collections are more likely to survive than for less senior scientists. In addition to trying to collect down the scientific hierarchies, we also wanted to more accurately reflect the gender balance. During the course of the HGAP we contacted twenty-eight female scientists, but so far have only managed to convert this into one small collection for the Wellcome Library, the Carol Churcher collection. Most of those who were spoken to had disposed of their material because they did not think that it would be of wider interest. The HGAP has ultimately also mainly focused on individuals rather than institutional records. Although this has not always been straightforward, it has been much easier to achieve that getting an organisation to preserve its records in a systematic way. For instance, EMBL first started thinking about establishing its own archive in December 2009 and aim to have their first archivist in post by the end of 2014.^39^ This process has taken a lot of hard work to build momentum and convince the organisation that this is worthwhile. I hope that this is part of a gradual change that will see archivists and scientists working more closely with each other in the future because the records of individual scientists alone will not accurately capture the full picture of how modern science is done.

## Footnotes

1 L. C. Dunn Papers held by The American Philosophical Society: http://www.amphilsoc.org/guides/glass/dunn.htm. There is a Guide to the Genetics Collections at the American Philosophical Society: http://www.amphilsoc.org/guides/glass/ (both accessed 8 May 2015).

2 At present accredited courses are offered by: Aberystwyth University; Northumbria University; University College Dublin; University of Dundee; University of Glasgow; University of Liverpool; and University College London. Archives and Records Association website: http://www.archives.org.uk/careers/careers-in-archives.html (accessed 8 May 2015).

3 See the Cold Spring Harbor Laboratory annual report for 2009, p.368: http://www.cshl.edu/images/stories/about_us/annual_reports/363-400BanburyCenter_09annrep.pdf (accessed 8 May 2015).

4 This paper focuses on the methodology used for the UK project. The other national projects have had to create their own methodologies in response to their own environments and challenges. For instance, the French project looks at contributions to the HGP from the start of the Centre d'Etude du Polymorphisme Humain's (Center for the Study of Human Polymorphisms) research on the human genome; the Japanese project looks at where Japan made significant contributions including work on individual chromosomes and the development of new technology; and the US project involves initiatives led by Cold Spring Harbor Laboratory and by the National Human Genome Research Institute. In November 2014 an initial meeting to establish a Chinese project was held.

5 For a more detailed account of the Royal Society/HMC pilot project see Ellis (1970).

6 Sir John Gaddum (reference JHG) and Sir Francis Simon collection (reference FS) both held by the Royal Society archives: http://www.aim25.ac.uk/cgi-bin/vcdf/detail?coll_id=5976&inst_id=18&nv1=browse&nv2=person (for Gaddum); http://www.aim25.ac.uk/cgi-bin/vcdf/detail?coll_id=5973&inst_id=18&nv1=search&nv2=person (for Simon). The Lawrence Wager collection was subsequently returned to the family before being deposited again. It is now held by Oxford University, Natural History Museum (reference **NCUACS 84.5.99):**http://discovery.nationalarchives.gov.uk/details/rd/7444c6ae-f937-4c12-b18a-196a1f827d04 (all accessed 8 May 2015).

7 NCUACS website preserved by the UK Web Archive: http://www.webarchive.org.uk/wayback/archive/20100204232541/http://www.bath.ac.uk/ncuacs/(accessed 8 May 2015).

8 Centre for Scientific Archives website: https://sites.google.com/site/centreforscientificarchives/(accessed 8 May 2015).

9 The Charity Commission holds the 2011 annual report for the Centre for Scientific Archives: http://apps.charitycommission.gov.uk/Accounts/Ends03/0001138303_ac_20111130_e_c.pdf (accessed 8 May 2015).

10 Even organisations at the forefront of working with born-digital material did not start doing this actively until the twenty-first century. For example, in the UK the Digital Preservation Coalition was founded in 2001 http://www.dpconline.org/about/dpc-history; the National Archives launched its first digital repository in 2003 http://www.nationalarchives.gov.uk/information-management/manage-information/preserving-digital-records/digital-repository/; and the Digital Curation Centre was launched in 2004 http://www.dcc.ac.uk/about-us/history-dcc/history-dcc (All accessed 8 May 2015).

11 Historians have also reflected on the problems that digital records pose for research and advocated for a more proactive approach to record-keeping. For two examples in this special issue see de Chadarevian (2016) and García-Sancho (2016).

12 Such as the Digital Preservation Handbook from the Digital Preservation Coalition: http://www.dpconline.org/advice/preservationhandbook/introduction (Accessed 8 May 2015).

13 There are several popular accounts of the HGP focusing on the race such as Davies (2002) and Shreeve (2007). These are balanced by more academically informed accounts including Cook-Deegan, 1994, Balmer, 1996, Fortun, 1998; and Bostanci (2004).

14 Wellcome Trust Sanger Institute overview of the sequencing of chromosome 22: http://www.sanger.ac.uk/about/history/hgp/chr22.html (Accessed 8 May 2015).

15 On the value of organisational collections kept by scientific administrators see García-Sancho (2016).

16 Archives and History Office at SLAC: http://www.slac.stanford.edu/history/;CERN archive:http://library.web.cern.ch/archives/CERN_archive (both accessed 8 May 2015).

17 EMBL mission: http://www.embl.de/aboutus/general_information/mission/index.html; EMBL archive: http://www.embl.de/aboutus/archive/index.html (both accessed 8 May 2015). On the parallelisms between scientific and archival practice see Peres (2016).

18 Bodleian Library, Saving Oxford Medicine collecting policy: http://www.bodleian.ox.ac.uk/bodley/finding-resources/special/projects/saving-oxford-medicine/collecting-policy (accessed 8 May 2015).

19 This assistance from the creator is recorded in the Wellcome Library catalogue for PP/COU: http://archives.wellcomelibrary.org/DServe/dserve.exe?dsqIni=Dserve.ini&dsqApp=Archive&dsqCmd=Show.tcl&dsqDb=Catalog&dsqPos=1&dsqSearch=%28Title%3D%27coulson%27%29 (Accessed 13 October 2014).

20 For instance see Ham (1975), p.8 and Samuels (1992), p.134.

21 In this attempt I was influenced by the work of historians looking at science from perspectives other than biography, such as de Chadarevian, 2002, Aicardi, 2014 and Bangham (2014).

22 UK National Archives custodial policy for digital archives, p. 6: http://www.nationalarchives.gov.uk/documents/information-management/custodial_policy.pdf (Accessed 13 October 2014).

23 It is not entirely clear why record-keeping is much more organised in the field of physics than in the field of biology. One theory is that physicists have been keener to demonstrate the value of their work and show that it extends beyond the destructive vision of the atomic bomb which has led them to think more about their historical legacy.

24 Research Resources in Medical History grant of £1500 to NCUACS, November 2008, GR087447, report p. 44: http://www.wellcome.ac.uk/stellent/groups/corporatesite/@policy_communications/documents/web_document/wtvm056353.pdf (Accessed 13 October 2014). The human genetics project at Cardiff saw the cataloguing and deposit of four main collections in established archives namely: John Edwards at Birmingham University Library (catalogue reference US99); George Fraser at the Wellcome Library (catalogue referencePP/GRF); Peter Harper at Cardiff University Special Collections and Archives (catalogue reference 609 PSH); and the MRC's Unit of Human Biochemical Genetics at UCL Archives (catalogue reference unknown).

25 Such as the Archives Hub: http://www.archiveshub.ac.uk/; Access2Achives (now part of Discovery through the advanced search facility): http://discovery.nationalarchives.gov.uk/a2a/; and the National Register of Archives (now part of Discovery through the advanced search facility): http://discovery.nationalarchives.gov.uk/(All accessed 8 December 2014).

26 Announcement of draft genome on the National Human Genome Research Institute website: http://www.genome.gov/10001356 (Accessed 13 October 2014).

27 This task was made easier by the new functionality on The National Archives' catalogue, Discovery: http://discovery.nationalarchives.gov.uk/ (Accessed 13 October 2014).

28 The Bishopsgate Institute holds protest and campaigning collections: http://www.bishopsgate.org.uk/content.aspx?CategoryID=1496 (Accessed 13 October 2014).

29 The stages in the Minnesota Method that were excluded by the HGAP were: defining functions performed by businesses and the collecting levels needed to document major functions; connecting documentary levels to priority tiers; and then updating and repeating the process every three to seven years. See Greene & Daniels-Howell (1997) for more detail on the Minnesota Method.

30 In this approach, I was encouraged by the work of historians such as Rebekah Higgitt: http://www.theguardian.com/science/the-h-word/2012/oct/03/history-science (Accessed 13 October 2014).

31 Material was found during surveys with people who worked in the robotics and automation team at the Sanger Institute. To date none of these collections have been deposited in archives and the survey reports remain confidential.

32 For instance, Sir Walter Bodmer was excluded because his papers had already been deposited in the Bodleian Library: http://www.bodleian.ox.ac.uk/bodley/finding-resources/special/projects/saving-oxford-medicine/catalogues-newly-available; material from the MRC is held by The National Archives (reference FD); the Towards Dolly project at the University of Edinburgh covers Edinburgh, Roslin and the birth of modern genetics: http://www.archives.lib.ed.ac.uk/towardsdolly/; and the UCL Genetics projects include geneticists who were based at UCL: http://www.ucl.ac.uk/library/about/projects (All accessed 13 October 2014).

33 LMB archives: http://www2.mrc-lmb.cam.ac.uk/about-lmb/archive-and-alumni/. The collections of former LMB directors Max Perutz and Aaron Klug and some senior scientists such as Cesar Milstein are at Churchill Archives in Cambridge: http://www2.mrc-lmb.cam.ac.uk/about-lmb/archive-and-alumni/manuscripts-correspondence/ (Both accessed 13 October 2014).

34 MRC collections held by The National Archives: http://discovery.nationalarchives.gov.uk/details/r/C121 (Accessed 13 October 2014).

35 When surveying HUGO's London office mostly published material was found, such as newsletters and annual reports, rather than original records, such as signed minutes of meetings.

36 Catalogue for the Sir Walter Bodmer collection: http://www.bodley.ox.ac.uk/dept/scwmss/wmss/online/modern/bodmer/bodmer.html (Accessed 13 October 2014).

37 The Bronwen Loder collection was acquired by the Bodleian in 2013 and included in their annual return to The National Archives: http://www.nationalarchives.gov.uk/accessions/2013/13digests/science.htm (Accessed 13 October 2014).

38 All collections held by the Wellcome Library have a catalogue entry that can be viewed through the Archives and Manuscripts catalogue, known as a collection level description:http://archives.wellcomelibrary.org/DServe/dserve.exe?&dsqIni=Dserve.ini&dsqApp=Archive&dsqDb=Catalog&dsqCmd=Search.tcl. Until a collection is catalogued it is not available to researchers, but the intention of these descriptions is to allow researchers to see the full extent of our holdings. Collections that have been catalogued can be ordered for viewing in the Rare Materials Room via the main Wellcome Library catalogue:http://wellcomelibrary.org/ (Both accessed 13 October 2014).

39 See EMBL's timeline for the process of establishing their archive: http://www.embl.de/aboutus/archive/timeline/index.html (Accessed 13 October 2014).
